# Serpentine Micromixers Using Extensional Mixing Elements

**DOI:** 10.3390/mi13101785

**Published:** 2022-10-20

**Authors:** George Tomaras, Chandrasekhar R. Kothapalli, Petru S. Fodor

**Affiliations:** 1 Department of Physics, Cleveland State University, 2121 Euclid Avenue, Cleveland, OH 44236, USA; 2 Department of Chemical and Biomedical Engineering, Cleveland State University, 2121 Euclid Avenue, Cleveland, OH 44236, USA

**Keywords:** passive micromixers, Dean flows and mixers, elongational flow, stretching flow, serpentine channels, hyperbolic constrictions, mixing index

## Abstract

Computational fluid dynamics modeling was used to characterize the effect of the integration of constrictions defined by the vertices of hyperbolas on the flow structure in microfluidic serpentine channels. In the new topology, the Dean flows characteristic of the pressure-driven fluid motion along curved channels are combined with elongational flows and asymmetric longitudinal eddies that develop in the constriction region. The resulting complex flow structure is characterized by folding and stretching of the fluid volumes, which can promote enhanced mixing. Optimization of the geometrical parameters defining the constriction region allows for the development of an efficient micromixer topology that shows robust enhanced performance across a broad range of Reynolds numbers from *Re* = 1 to 100.

## 1. Introduction

Microfluidic systems have evolved as an important support platform for a vast range of applications in the chemical and biological sciences, from biological/chemical analysis [1,2] to reaction engineering and the development of organ-on-chip technologies [3,4]. Their success lies in their potential for effective operation with limited consumption of reactants, high rates of heat and mass transfer, precise control over reaction variables such as temperature and concentrations, as well as providing the ability to manipulate particles, biologically relevant chemical systems, and cells [5,6]. Most of their applications require the homogenization of two or more chemical and/or biological species, making mixing a critical functional requirement. However, the flow in the regime of low Reynolds numbers (Re) encountered in many proposed applications is laminar; thus, the mixing on these scales is diffusion-dominated, which is unfortunately slow [7,8,9].

Achieving efficient mixing requires the presence of vortices and the folding/stretching of fluid volumes. These are the basic phenomena through which the interface between the species to be mixed is expanded, to increase the effective area for diffusion, and through which the particles and chemical species of interest are dispersed throughout the volume of the flow [10,11]. To this end, in microfluidic systems, researchers have employed both active and passive strategies to induce the desired vortices and flow structures. Active micromixers use external energy sources to generate the necessary stirring motion [12]. Methodologies include the use of acoustic waves [13,14], magnetic interactions [15,16,17], laser-induced bubble formation [18], and electric interactions [19]. While their efficiency can be high, they can be complicated to design and manufacture [12].

Passive micromixers, on the other hand, rely only on appropriately designed geometrical structures placed in the path of the flow driven by a pressure gradient to manipulate the fluid motion. The geometrical structure of the system is designed to either split and recombine the fluid streams multiple times in order to increase the interfacial surface area between the fluid components and ultimately enhance the diffusional mixing efficiency or generate complex flow structures that promote chaotic advection. While high-performance split-and-recombine passive mixers have been implemented [20,21,22], their geometry is quite complex, making their manufacture challenging. Thus, in recent decades, a large body of work has been dedicated to the development of passive mixers seeking to promote chaotic advection, as they can achieve good performance while employing less-demanding topologies [23]. 

Generally, in passive systems, achieving good mixing requires the fluid volumes to be subjected to rotational cross-sectional flows that promote crosstalk between the fluid streams that carry the different chemical/biological species, as well as extensional flows [10], leading to sequences of folding and stretching of the fluid elements. Methodologies to achieve cross-sectional flows include: the placement of slated groove–ridge systems across the bottom or top of the channel, forcing the axial pressure gradient to drive transversal flows [24,25]; adding curved sections to the channel (spirals, helixes, or serpentines) and relying on the centrifugal forces experienced by the fluid to drive the transversal flows (i.e., Dean vortices) [23,26]; or using a combination of both [27]. Development of extensional flows in groove–ridge systems is achieved by using asymmetric grooves either organized in periodic half-cycles, such as in the well-known staggered herringbone configuration [25,28,29], or generated quasi-randomly using a fractal algorithm [30,31]. These result in local extensional flows associated with the shift in the cross-sectional vortices’ centers of rotation. In curved/serpentine channels, a similar local stretching of the fluid elements has been achieved through a variety of methods. Sayah and Gijs [32] have employed mixing units composed of multiple curved sections situated in orthogonal planes that force the fluid to undergo 3D turns. This results in a rapid expansion of the interface between the components to be mixed and thus increases the efficiency of the molecular diffusion. Hossain and Kim [33] proposed the use of non-aligned inputs at the T-junction inlet of a serpentine channel. They show that this initial asymmetry generates a vertical vortex that increases the complexity of the transversal flow circulation and is conducive to enhanced mixing. The use of L-shaped non-rectangular cross-sections has been exploited by Clark et al. [34] as a way of controlling the position of the transversal centers of rotation for the Dean vortices. This results in the stretching of the fluid elements between the mixing units and leads to flow structures similar to those found in staggered herring-bone-type systems, thereby greatly increasing the chaotic advection component of the mixing. Usefian and Bayareh [35] have developed a mixing strategy based on convergent–divergent sections that force the fluid through sequences of compressions and expansions. While Dean vortices are not formed in their configuration, due to the low aspect ratio of the curved sections, the expansion vortex that forms due to the presence of the convergent–divergent sections leads to effective mixing performance for a broad range of Reynolds numbers. The idea of using convergent–divergent sections has also been exploited by Afzal and Kim [36]. Their design employing sine functions to define the boundaries of the channel combines the effect of the Dean flows present in subchannels and the split–recombination of the flows in the main channel sections. In serpentine micromixers, the complex flows induced by mixing can also be achieved by operating at high Reynolds numbers Re > 100, where flow bifurcation and the consequent formation of multiple cross-sectional vortices occurs [37]. However, the pressure differentials required limit the practical feasibility of operating within these conditions. 

**Figure 1:**
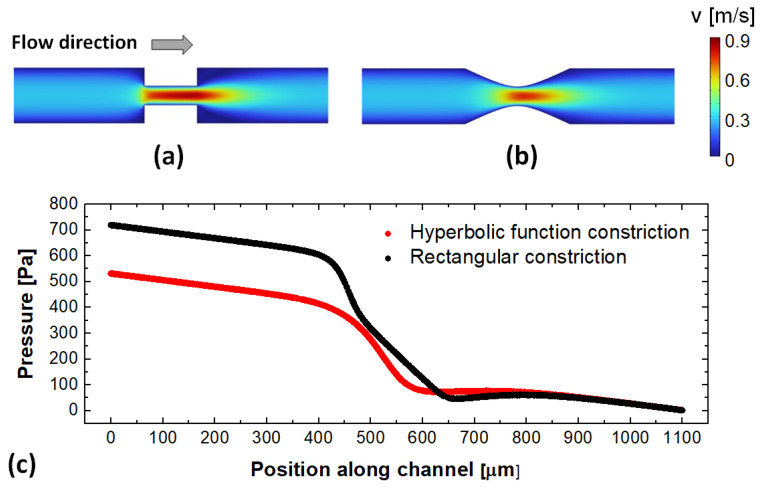
Velocity field maps along the center plane for: (**a**) a channel with a rectangular constriction and (**b**) a channel with a constriction defined by a hyperbolic function, respectively. (**c**) Corresponding pressure gradient profiles along the two channels, showing lower pressure drops for the same constriction diameter for the hyperbolic design.

In the current work, we propose and evaluate the performance of a micromixer in which semi-circular curved sections in a serpentine channel are used to generate cross-sectional flows, while elongational flows are achieved via the inclusion of constrictions defined by hyperbolic functions between the adjacent curved sections.. The inspiration for the use of constrictions to increase the mixing in laminar systems originates from the work of Carson et al. on the mixing of polymers by utilizing converging and diverging flows achieved through hyperbolic obstacles [38]. In those particular laminar systems, the large velocity gradients and associated extensional flows have shown robust mixing across a broad range of flow conditions and viscosities. As described in Section 2, using hyperbolic functions allows for the geometrical parametrization of the designs with specific parameters controlling the length and width of the constriction. Moreover, using this particular geometry for the constriction allows one to obtain similar velocity gradients in the flow, but at lower pressure drops across the constriction (Figure 1—data obtained using the numerical methods as described in Section 3). As discussed in Section 4, the pairing of the Dean flow characteristics of fluid motion through curved channels with the elongational flows associated with the constriction enhances the mixing capabilities in these microscale systems.

## 2. Geometrical Design of the Micromixer

**Figure 2:**
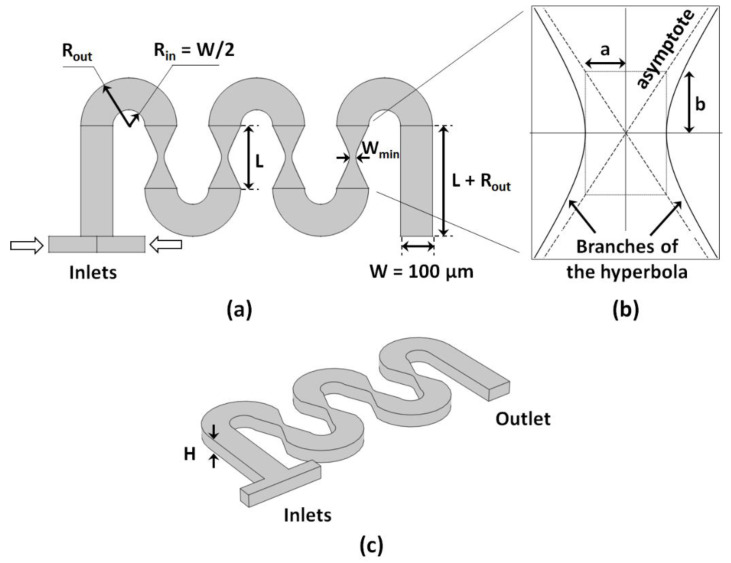
(**a**) Top view of the geometry of the investigated design; (**b**) hyperbola defining the constriction of the straight sections between adjacent curves; (**c**) 3D geometry of the channel investigated.

The serpentine design used in this study consisted of rectangular cross-sections of width *W* = 200 μm and height *H* = 100 μm, and semi-circular curved sections of inner radius *R_in_ = W/2* and outer radius *R_out_ = R_in_ + W* (Figure 2). Adjacent curved sections are connected by constrictions defined by hyperbolas generated using the following equation:

(1) $$\frac{x^{2}}{a^{2}}-\frac{y^{2}}{b^{2}}=1$$

where the parameter *a* corresponds to the half-distance between the vertices of the branches of the hyperbola, and the parameter *b* corresponds to half of the height of the rectangle whose diagonals are the asymptotes of the hyperbola. Hence, *a* determines *W_min_ = 2a*, i.e., the minimum width of the constriction, and *b* determines L, i.e., the length of the constriction. Straight sections of length *L + R_out_* are placed at the entry and exit of the mixer. The fluids to be mixed are pumped into the system through two inlets of width *W/2*. 

The geometry of the constriction is parametrized by setting the following parameter values: *a* = {20, 35, 50, 65, 80} [μm], with *b* spanning the range *b* = {0.5*a*, 0.75*a*, 1.0*a*, 1.5*a*, 2.0*a*, 2.5*a*, 3.0*a*, 4.0*a*, 5.0*a*} (Table 1). The performance of the designs with constrictions was compared with that of serpentine channels of the same length, in which the constriction region was replaced with a straight section of equal length.

## 3. Numerical Modeling and Assessment of Mixing

The steady-state Navier–Stokes equations for an incompressible Newtonian fluid are solved to determine the flow field in the microchannels investigated:

(2) $$\rho \left(v\cdot \nabla \right)v=-\nabla p+\eta \nabla^{2}v$$

(3) ∇·v=0

where v [m∙s^−1^] is the velocity vector, *ρ* [kg∙m^−3^] is the fluid density, *η* [kg∙m^−1^∙s^−1^] is the fluid viscosity, and *p* [Pa] is the pressure. The values for the density and viscosity were set to those for water at room temperature, i.e., *ρ* = 10^3^ kg∙m^−3^ and *η* = 10^−3^ kg∙m^−1^∙s^−1^. No-slip boundary conditions were set for all of the side walls of the micromixers, while the outlet was set at zero pressure. Once the flow fields are mapped, they were used as inputs for solving the concentration–diffusion equation to determine the concentration *c* [mol∙m^−3^] evolution through the volume of the micromixers:

(4) $$v\cdot \nabla c=D\nabla^{2}c$$

where *D* [m^2^∙s^−1^] is the diffusion coefficient. This is set to 1.0 × 10^−9^ m^2^∙s^−1^_,_ corresponding to the typical diffusivity range of most ions in aqueous solutions.

To quantify the quality of mixing, the molar concentration for the fluid entering one of the inlets is set to *c* = 1 mol∙m^−3^, while for the opposite inlet it is set to *c* = 0 mol∙m^−3^. The mixing index is quantified based on 8-bit grayscale snapshots of the concentration distribution across the micromixer’s outlet (maximum intensity = 255 corresponding to *c* = 1 mol∙m^−3^ and minimum intensity = 0 corresponding to *c* = 0 mol∙m^−3^, respectively). The mixing index (*M*) is calculated using a procedure based on the Shannon information entropy of concentration maps [28]. Specifically, for a two-component system, the mixing index is given by:

(5) $$M=-\frac{1}{ln\left(2\right)}\cdot \frac{1}{N_{bins}}\cdot \sum_{j=1}^{N_{bins}}\left[p_{1/j}ln\left(p_{1/j}\right)+p_{2/j}ln\left(p_{2/j}\right)\right]$$

where *N_bins_* is the number of bins in which the image is partitioned; and $p_{1/j}$ and $p_{2/j}$=$1-p_{1/j}$ (since we are working with two components and the fluids are incompressible) are the conditional probabilities of the two components to be located in bin *j*. The latter are calculated directly based on the intensity of the concentration images. The term ln(2) normalizes the mixing index based on the fact that there are two components. Under the above definition, *M* is easy to interpret as it takes values between 0 and 1, corresponding to completely segregated (no mixing) and completely mixed components (ideal mixing), respectively.

The simulations for both the flow fields and concentration distributions were performed using the COMSOL Multiphysics Computational Package (COMSOL, Inc., Burlington, MA, USA) and its Computational Fluid Dynamics and Chemical Engineering modules. For all simulations, a free unstructured tetrahedral mesh was used with typical mesh elements with an edge no larger than 12.5 μm for the flow field solver, and no larger than 7.8 μm for the concentration solver, respectively. The higher resolution mesh size is required when solving the concentration–diffusion equation, given the small scales associated with diffusional mixing. The mesh is chosen by performing a grid validation study. This is to ensure that a sufficiently fine mesh is used to achieve high accuracy and limit erroneous readings in the mixing performance due to numerical artificial diffusion [39], while keeping the simulations computationally efficient. Additionally, the computational models used have been previously validated against experimental data from similar serpentine type systems [40].

## 4. Results and Discussion

**Figure 3:**
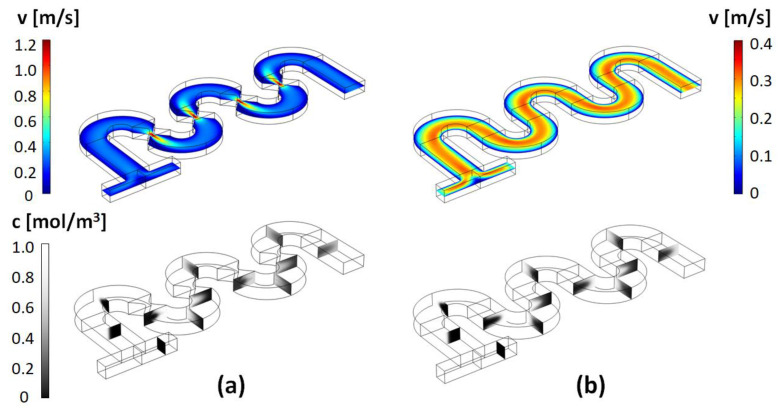
(**top**) Longitudinal cross-section at *H/2* (middle of the channel) of the magnitude of the velocity map and (**bottom**) concentration cross-sectional maps along the channel, for: (**a**) the design with *a =* 20 μm and *b* = 1 × *a*; and (**b**) the corresponding simple serpentine channel (Reynolds number *Re* = 20).

**Figure 4:**
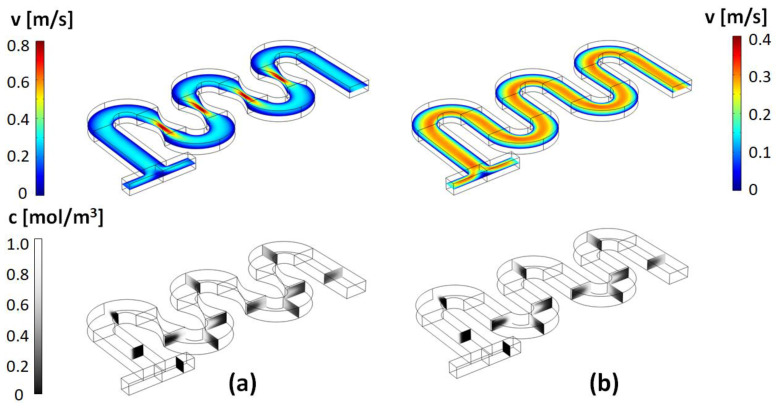
Velocity magnitude and concentration maps for the (**a**) design with *a =* 35 μm and *b* = 2 × *a*; and (**b**) the corresponding simple serpentine channel (*Re* = 20).

**Figure 5:**
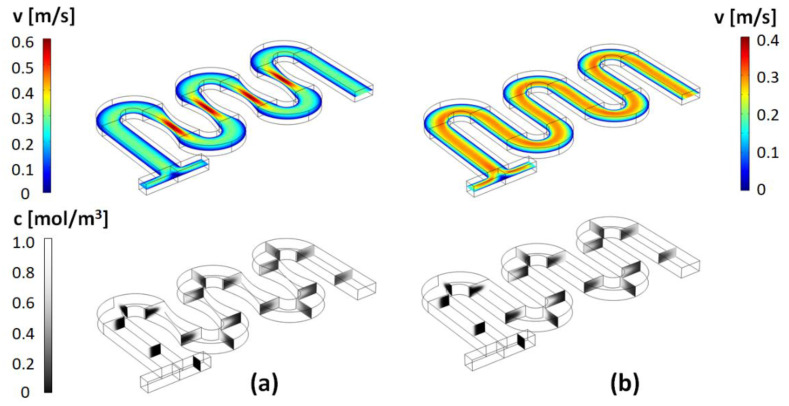
Velocity magnitude and concentration maps for the (**a**) design with *a =* 50 μm and *b* = 3 × *a*; and (**b**) the corresponding simple serpentine channel (*Re* = 20).

Typical results for the flow fields and concentration distribution in this type of micromixer, as well as for the corresponding un-constricted serpentine micromixers, are shown in Figure 3, Figure 4 and Figure 5. As described above, a broad set of designs are characterized based on the parametrization of the geometrical parameters *a* and *b.*

**Figure 6:**
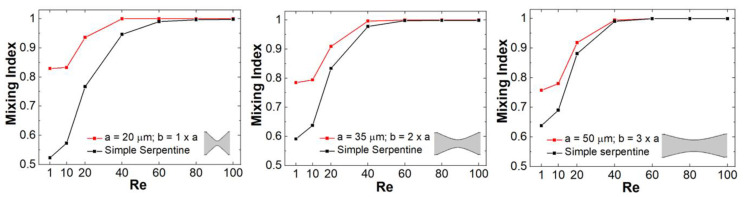
Reynolds number dependence of the mixing index of mixers with: (**left**) *a =* 20 μm and *b* = 1 × *a*; (**middle**) *a =* 35 μm and *b* = 2 × *a*; and (**right**) *a =* 50 μm and *b* = 3 × *a*.

As expected, based on previous studies of spiral, helical, or serpentine systems that contain curved channel sections, the centrifugal interactions experienced by the fluid led to the development of cross-sectional Dean flows [23]. These result in the observed C-shaped stretching of the interface between the two fluid components and their transversal circulation as they move along the channel. However, at low Reynolds numbers, and in simple serpentines, while some mixing is associated with this effect, the two components remain largely separated in distinct regions across the transversal planes of the channel. On the other hand, visually, in the channels designed with constrictions, the evolution towards homogeneity of the concentration profiles appears much more rapid. This can be quantified using the mixing index defined in Equation (5). Figure 6 gives a comparison of the mixing index in constricted channel geometries relative to their simple serpentine counterparts for Reynolds numbers taking values of *Re* = 1, 10, 20, 40, 60, 80, and 100 (corresponding to inflow mean velocities from 0.0075 m/s to 0.75 m/s). Table 2 lists the corresponding pressure drops and Dean numbers for this simulation set ($De=Re\cdot \sqrt{\frac{D_{h}}{2\cdot R_{mid}}}$, where *D_h_* is the hydraulic diameter of the channel and $R_{mid}=\frac{3}{4}W$ is the radius of curvature corresponding to the central path of the curved sections, respectively).

**Figure 7:**
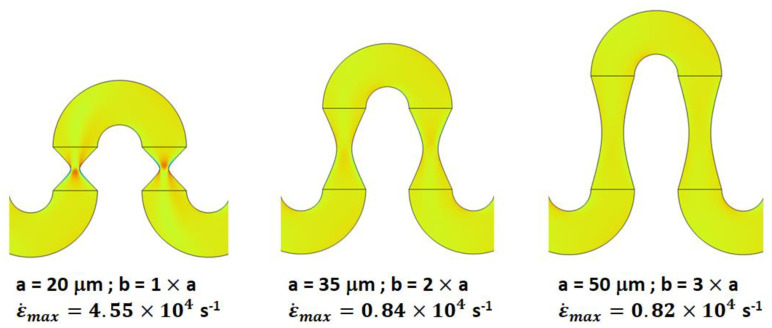
Stretch rate maps along the longitudinal cross-section of various constricted serpentine channels studied in this work. Insets specify the geometrical parameters of the channels, as well as the maximum value ${\dot{\varepsilon }}_{max}$ of the ${\dot{\varepsilon }}_{y}$ stretch rate observed (*Re* = 20).

For all the Reynolds numbers investigated, the performance of the new mixer design exceeds or matches that of simple serpentines, indicating that more complex flow structures are generated in these designs. Particularly, based on the flow velocity magnitude dependence along the channel (Figure 3, Figure 4, Figure 5), one of the distinctive features of the new type of micromixer is the presence of large velocity gradients in the constriction region which would expose the fluid elements to large elongational flow components. One way to quantify this is to compute the stretch rate along the longitudinal axis of the channel ${\dot{\varepsilon }}_{y}$ [41]:

(6) $${\dot{\varepsilon }}_{y}=\frac{dv_{y}}{dy}$$

where $v_{y}$ is the component of the velocity vector along the longitudinal axis of the channel, which for our geometry orientation is the y-axis. Figure 7 shows a comparison between the elongational flow experienced by the fluid for constrictions with different *a* and *b* parameters. Not unexpectedly, the strength of the stretch rates is maximized for narrow and short constrictions. More importantly though, the stretch rates experienced by the fluid are correlated with the values of the mixing indexes achieved, highlighting the importance of elongational flows in achieving high mixing quality. 

**Figure 8:**
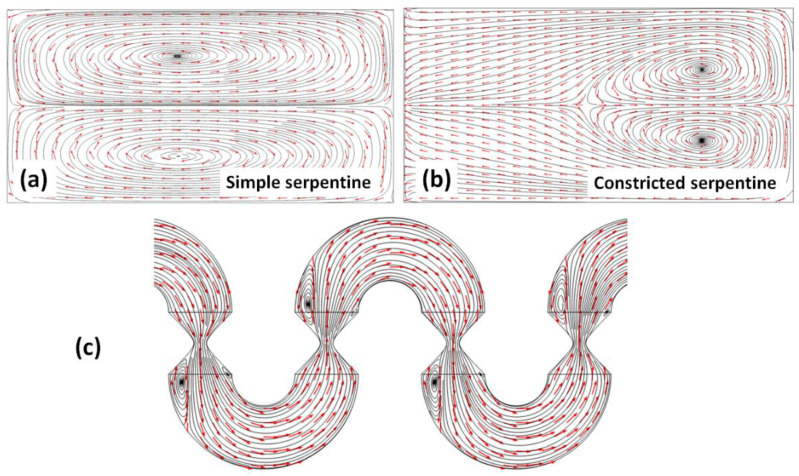
Streamline plots for: (**a**) transversal section in a serpentine channel; (**b**) transversal section in a constricted channel; and (**c**) longitudinal section in a constricted channel (*Re* = 40).

In order to gain insight into the nature of the flows in this type of channels, streamline plots were constructed from the solutions to the Navier–Stokes equations for both the transversal and longitudinal sections across the channel. As Figure 8 indicates, the nature of the flows developing in the channels with constrictions is strikingly different from that of flows in simple serpentines. Transversal cross-sections of the streamline plots in simple serpentines indicate the presence of counter-rotating vortices centered on the top half and the bottom half, respectively, of the channel (Figure 8a). These vortices, i.e., the Dean flows, are responsible for the initial stretching and subsequent folding of the interface between the two components to be mixed, and for why this type of design has attracted interest in passive micromixing applications. While a necessary condition for mixing, the presence of transversal flows is insufficient for achieving rapid mixing. In the simple serpentine, the cross-sectional flows are symmetric, with little change in the centers of rotation of the transversal flows between mixing units. In the constricted serpentines however, the symmetry of the flows relative to the vertical symmetry axis of the cross-section is broken (Figure 8b). The transversal flow profiles are more complex and, more importantly, between mixing units, the positions of the centers of rotation shift from one side of the channel to the opposite one. Thus, in this geometry, the fluid is subjected to repeated sequences of folding (within the curved mixing units) and stretching (within the constrictions). This type of flow structure has been encountered in other systems where a high quality of mixing is achieved, such as the staggered herringbone groove–ridge design [25], serpentine mixers with non-rectangular cross-sections [34], or serpentine mixers operated at very high Reynolds numbers [23]. Additionally, in the particular system investigated, longitudinal streamline plots indicate the formation of longitudinal eddies associated with the expansion of fluid past the constriction. Each constriction is associated with two longitudinal eddies, asymmetric in size, with the larger one on the outer region of the flow, that further contribute to the redistribution of the fluid components within the volume of the channel.

**Figure 9:**
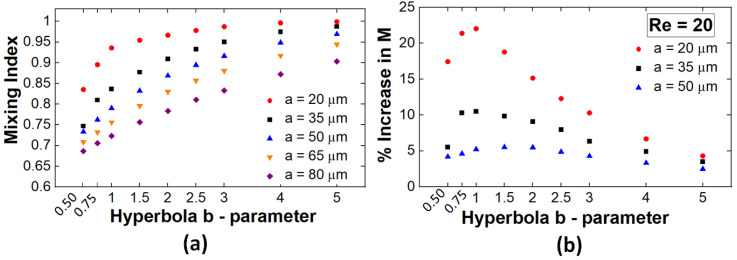
(**a**) Parametric study of the mixing index dependence on the *a* and *b* parameters (*Re* = 20); (**b**) increase in the mixing performance relative to simple serpentine designs.

The above observations provide qualitative insights into the flow structures that develop in these geometries and how they are able to enhance mixing performance. In order to determine the specific geometrical parameters where the effect of this type of flows is maximized, a full parametric study for *a* = {20, 35, 50, 65, 80} [μm] and *b* = {0.5a, 0.75a, 1.0a, 1.5a, 2.0a, 2.5a, 3.0a, 4.0a, 5.0a} has been performed. Based on the results presented in Figure 9a, at a fixed inflow speed, the maximum mixing performance is achieved in channels with narrow (small *a*) and long (large *b*) constrictions. Later observations seem to run counter to the previous conclusion that large stretch rates that are associated with short constrictions are associated with increased mixing. Nevertheless, it has to be noted that longer constrictions are also geometrically associated with longer channels and thus longer residence times. Consequently, some of the increased mixing performance at larger *b* values is associated with longer times for the diffusion to act on the components to be mixed. To account for this effect, in Figure 9b, the mixing efficiency as a function of the *b* length of the constriction, is normalized by the performance of the corresponding simple serpentine channels. For each *a* value set, a clear maximum can thus be identified in the lower range of *b* values. Thus, these data are consistent with the expectation that, as the length of the constriction increases and the stretch rates consequently decrease, the mixing performance converges to values similar to those observed in unmodified designs. The procedure then allows for the optimization of the constriction geometrical parameters to achieve high-quality mixing while minimizing the needed constriction length and thus maximizing the mixing performance achievable per unit length in this design.

## 5. Conclusions

In the present study, we investigated a new design for a serpentine micromixer with semi-circular curved sections employing constrictions formed by the vertices of hyperbolas in the connecting sections. The typical Dean flow structures present in curved channels are complemented in this design by elongational and backstep flows that develop in the constriction regions. Quantitative assessment of the mixing demonstrated enhanced performance in these mixers relative to simple serpentine mixers, across all Reynolds numbers investigated, with optimized designs achieving reliable mixing values better than 0.93 within two mixing cycles for *Re* > 20. In this work, the constrictions were used as a mixing-enhancement strategy for serpentine channels; however, it is important to note that this type of flow modification strategy can be easily integrated within other mixing designs that achieve transversal flows, through different means, such as groove–ridge systems.

## Figures and Tables

**Table 1:** List of the combinations of (*a*, *b*) geometrical parameters used in the simulations. All dimensions are listed in micrometers [μm].

	b	0.5 × a	0.75 × a	1.0 × a	1.5 × a	2.0 × a	2.5 × a	3.0 × a	4.0 × a	5.0 × a
a										
**20 μm**		(20, 10)	(20, 15)	(20, 20)	(20, 30)	(20, 40)	(20, 50)	(20, 60)	(20, 80)	(20, 100)
**35 μm**		(35, 17.5)	(35, 26.25)	(35, 35)	(35, 52.5)	(35, 70)	(35, 87.5)	(35, 105)	(35, 140)	(35, 175)
**50 μm**		(50, 25)	(50, 37.5)	(50, 50)	(50, 75)	(50, 100)	(50, 125)	(50, 150)	(50, 200)	(50, 250)
**65 μm**		(65, 32.5)	(65, 48.75)	(65, 65)	(65, 97.5)	(65, 130)	(65, 162.5)	(65, 195)	(65, 260)	(65, 325)
**80 μm**		(80, 40)	(80, 60)	(80, 80)	(80, 120)	(80, 160)	(80, 200)	(80, 240)	(80, 320)	(80, 400)

**Table 2:** Dean numbers, *De*, and pressure differentials, Δ*P*, for mixers with various (*a,b*) geometrical parameters across the Reynolds numbers investigated.

*Re*	1	10	20	40	60	80	100
(*a* = 20 μm, *b* = 20 μm)	*De* = 0.66 Δ*P* = 0.167 kPa	*De* = 6.6 Δ*P* = 1.76 kPa	*De* = 13.3 Δ*P* = 3.93 kPa	*De* = 26.6 Δ*P* = 10.1 kPa	*De* = 40 ΔP = 18.8 kPa	*De* = 53.3 Δ*P* = 30.0 kPa	*De* = 66 Δ*P* = 43.6 kPa
(*a* = 35 μm, *b* = 70 μm)	*De* = 0.66 Δ*P* = 0.135 kPa	*De* = 6.6 Δ*P* = 1.38 kPa	*De* = 13.3 Δ*P* = 2.85 kPa	*De* = 26.6 Δ*P* = 6.33 kPa	*De* = 40 Δ*P* = 10.7 kPa	*De* = 53.3 Δ*P* = 15.9 kPa	*De* = 66 Δ*P* = 22.0 kPa
(*a* = 50 μm, *b* = 150 μm)	*De* = 0.66 Δ*P* = 0.130 kPa	*De* = 6.6 Δ*P* = 1.32 kPa	*De* = 13.3 Δ*P* = 2.68 kPa	*De* = 26.6 Δ*P* = 5.69 kPa	*De* = 40 Δ*P* = 9.2 kPa	*De* = 53.3 Δ*P* = 13.3 kPa	*De* = 66 Δ*P* = 17.9 kPa
